# Human Health Consumption Risk Assessment of Trace Metal Content in the Triggerfish *Balistes* spp. from the RAMSAR Site 1826 San Ignacio-Navachiste-Macapule Lagoon Complex

**DOI:** 10.3390/toxics13090718

**Published:** 2025-08-27

**Authors:** Héctor Abelardo González-Ocampo, Adán Alfonso Michel-Rubio, Ernestina Pérez-Gonzalez, Guadalupe Durga Rodríguez-Meza

**Affiliations:** 1 Instituto Politécnico Nacional, CIIDIR Unidad Sinaloa, Juan de Dios Bátiz Paredes S/N, Col. San Joachín, Guasave 81106, Sinaloa, Mexico; eperezg@ipn.mx; 2 Universidad Autónoma de Occidente, Campus Guasave, Avenida Universidad S/N, Flamingos, Guasave 81048, Sinaloa, Mexico; adan_mr10@hotmail.com

**Keywords:** fish, food safety, Navachiste, pollution, risk, trace metal

## Abstract

Trace metal (TM) concentrations and carcinogenic risk were determined in ninety-two edible samples of the triggerfish *Balistes* spp. from the RAMSAR site 1826 San Ignacio-Navachiste-Macapule Lagoon Complex (NAV). The acid digestion method and an atomic absorption spectrophotometer were used to determine TM concentrations. Calibration curves were carried out using TORT-3 reference materials. The blank and certified reference materials were treated using the same procedure as a sample. TM sequence was Zn > Fe > Mn > Cu = Pb > Ni > Cd, and a correlation (*p* = 0.0169) between size and concentrations was found. No correlation (*p* = 0.079) was found between weight and concentrations, or sampling sites and the concentrations. The highest concentrations were found during the summer, followed by the spring and winter of 2017, while the lowest was found in the winter of 2018. The Zn was significantly higher in summer-17. The Target Hazard Quotient (THQ) was <1 for Pb, Ni, Cd, Fe, Zn, and Cu, and 1.39 for Mn. Cd and Pb resulted in carcinogenic potential (CsFo < 1) with a very low probability. The TM concentrations and bioaccumulation in triggerfish showed no consumption risk, due to its omnivorous diet, and trophic transfer rates were described for aquatic food webs.

## 1. Introduction

Among the most important anthropogenic activities that generate trace metal (TM) residues are metallurgy, the leather industry, vehicle emissions, aquaculture and intensive agriculture [1]. Coastal lagoon pollution caused by anthropogenic sources includes organic and inorganic compounds affecting the ecosystems and the inhabiting biota. RAMSAR site 1826 San Ignacio-Navachiste-Macapule Lagoon Complex (NAV) is a shallow coastal lagoon located in the southeastern part of the Gulf of California. Among the anthropogenic sources of TM are the residues of the fertilizers and pesticides due to intensive application in aquaculture, agriculture and urban activities, whose residues are discharged to this lagoon [2]. A large part of these residues is mostly transported by erosion and rain and dragged through discharge channels to the coastal ecosystems [3], so they may occur in NAV. In the water column, TM residues adhere to organic matter, and marine sediments efficiently trap chemical elements suspended in the water column [4]. Thereafter, the biomagnification and bioaccumulation processes begin, and through the trophic web, TMs becomes among the most important pollutants bioaccumulated in the edible tissues of marine species [5].

One of the more profitable fisheries in the Gulf of California is the finescale triggerfish fishery [6]. The Gulf of California is characterized by its biological diversity of marine flora and fauna and the RAMSAR site 1826 San Ignacio-Navachiste-Macapule Lagoon Complex (NAV) spans more than 28,000 ha, semi-surrounded by intensive aquaculture and agriculture located at the southeastern part of this gulf [2]. Previous studies in NAV have determined the presence of TM in the sediments [7,8] and edible tissues of marine species inhabiting this lagoon [9,10,11,12]. 

The fine-scale triggerfish *Balistes polylepis* inhabits rocky reefs, boulder-strewn slopes and sandy areas. In is demersal in adulthood and pelagic when young [13], and feeds on marine invertebrates such as sea urchins, crustaceans and mollusks [14]. This species has come to be an important fishery resource and a steady income for the artisanal fisherman in the Gulf of California [15,16]. This species is a demersal fish that inhabit sandy–muddy bottoms [17], such as those described for the NAV, and due to its feeding habits and its trophic position, its trace metal content has been reported [15,16,18,19].

Despite the significance of *B. polylepis* in artisanal and commercial fishing, no more information has been published in the last 17 years regarding the concentrations of TM in this species, and even less about the health risks associated with its consumption. In NAV, this species provides substantial income for local fishermen, and its commercial consumption in the region has increased over the past decade. Given the increasing consumption rate in Mexico, the present study primarily aims to determine the concentration of TMs in the fillet of *B. polylepis* captured from NAV, and subsequently, evaluate the human health risks associated with its consumption.

## 2. Materials and Methods

### 2.1. Study Area

**Figure 1:**
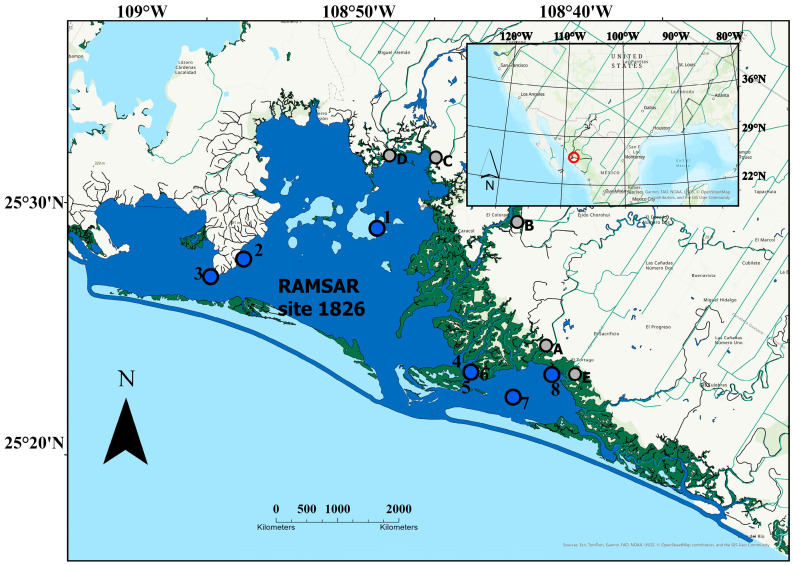
Sampling points (blue numbered dots) and discharge agricultural drain locations (grey alphabetic dots) in the RAMSAR site 1826 San Ignacio-Navachiste-Macapule Lagoon Complex. 7 = macapule; A = Sartajoa drain; B = San Antonio drain; C = Batamote drain; D = Navobampo; E = El Tortugo Drain.

NAV is a coastal lagoon complex (~24,000 Ha) located in the southeastern part of the Gulf of California (25.4°–25.7° N and 108.85°–108.55° W) [20]. Since 1978, NAV islands have formed part of the Natural Protected Area Gulf of California Islands (ANP), and in 2008 NAV was declared as RAMSAR site 1836 [21] to become a location of economic importance and ecology management priority. This ecosystem is considered a shallow lagoon due to its average depth (2.5 m) between 0.5 and 5.0 m, having deepest zones up to 11 m near the mouths [20,22] (Figure 1). Eight sampling sites were selected based on the traditional fishing points according to the season of the year: Macapule, Relices, Ventana, Taikori, Travesaño, Meroboca, Isla Pájaros, and Vasiquilla.

### 2.2. Sample Collection

With the assistance of a local fisherman with a valid fishing permit issued from National Fisheries Commission, which is the agency in charge of regulation and issuing licenses of fishing and aquaculture permits [23], ninety-one specimens of *Balistes* spp. trigger fish were captured during four collecting periods, on 5 May 2017 (4), 8 August 2017 (35), 12 December 2017 (5), 11 January 2018 (25), and 1 March 2018 (22). Fish were collected seasonally in the NAV using a fishing net. The biometric data (height and weight) were recorded in the field. The specimens were sacrificed according to legal requirements or guidelines in Mexico for the care of animals, and captured fishes were acquired with a fisherman possessing an official fishing permit. Samples were transported to the Organic and Inorganic Contamination Laboratory at the CIIDIR-IPN Sinaloa Center where the samples were kept in a refrigerator at 4 °C for later processing. Muscles and organs were previously removed from each specimen to be subsequently dissected. The remaining samples were placed in plastic bags inside porcelain capsules and dried at 90 °C for 72 h.

### 2.3. Sample Preparation

To avoid alterations in the results of trace metal concentrations, the plastic and glass material was washed with phosphate-free soap and rinsed with running water. It was then immersed in hydrochloric acid (FAGALAB^®^, Cod. 2024 Mocorito, Mexico), for 24 h and rinsed with nitric acid (FAGALAB^®^, Cod 2036 Mocorito, Mexico). After washing the material, it was rinsed with deionized water (FRAMAR^®^ Niagara Falls, ON, Canada) and left to dry in a clean, metal-free environment. Once dry, it was stored in a plastic wrap with blotting paper and cellophane. The seawater pH and temperature were determined based on published literature [24,25].

### 2.4. TM Analysis

Detection and quantification of TM were performed by the standard acidic digestion method of the muscles [26]. A total of 0.5 g of dry and homogenized muscle tissue was weighed on an analytical balance (OHAUS^®^ Analytical Plus, Mumbai, India), and placed in a 500 mL Erlenmeyer flask on a heating plate, and 5 mL of analytical grade nitric acid (HNO_3_) (FAGALAB, Cod 2036, Mocorito, Mexico) was added into the flask for total digestion. The flasks were covered with watch glasses to prevent the escape of sample vapors.

Muscle samples were set with the acid to react for 4 h until a clear solution was obtained. After the digestion period samples were allowed to cool at room temperature. The resulting solution was placed into a 50 mL flask with deionized water (FRAMAR^®^, Niagara Falls, ON, Canada). A funnel was used to reduce the number of solid residues to avoid interferences in the Atomic Absorption Spectrophotometry analysis. All resulting solutions from acidic digestions were stored in 50 mL polypropylene tubes.

The TM concentrations were determined by triplicate samples following the method NOM-117-SSA1-1994 [26], using atomic absorption spectrophotometry (AAS) (AVANTA GBC^®^, Shelton, CT, USA) with an air-acetylene flame burner with hollow cathode lamps. The wavelengths (nm) to determine the TMs in the samples were 228.8, 324.8, 248.3, 285.2, 232, 213.9, and 283.3 for Cd, Cu, Fe, Mn, Ni, Zn, and Pb, respectively. TM standards (Table 1) curves were obtained for certified material (none of the certified material had reached its expiry date) dissolutions (0.125, 0.25, 0.5, 1, 2, and 4 ppm) to ensure the accuracy of the AAS. Blanks were analyzed after ten edible samples to validate the results. Verification of the method, sample handling, cleanliness of the material, and quality of the reagents were constantly checked. Results were described as mean ± standard deviation. The blanks were treated as a sample under the same method, recovering a percentage of each element following the equation below:

(1) $$TM\;recovery\;(\%)=\frac{TM\;Certified\;value\;(mg/kg)}{Resulted\;TM\;(mg/kg)}\times 100$$

Subsequently, the reading was carried out in the AAS, which begins by optimizing the equipment specifications, such as air and acetylene flow, burner height, slot width, and lamp wavelength for each metal used. After the stabilization time, the calibration curve of each element was read, considering seven calibration points with an average correlation coefficient above 0.99 to ensure accuracy in the analysis of samples and data analyzed in the laboratory. Due to the high content in the samples, Fe, Zn, and Mn were diluted with deionized water.

For the concentration of TMs, a linear regression equation (Equation (2)) in a Microsoft Excel spreadsheet was carried out [26] using the following equation:

(2) Y=ax+b

where:

*Y* is the absorbance of the sample; *a* is the slope (absorptivity coefficient); and *b* is the ordinate at the origin.

To obtain the concentration, *x* was moved to the other side, and the volume of the digested sample (50 mL) and its dry weight were recorded.

### 2.5. Estimated Daily Intake (EDI)

*EDI* is the proximal ingestion that a person ingests daily over an exposure period to establish the analyte concentration. The *EDI* was calculated based on the metals analyzed [27,28] with the following formula [28]:

(3) $$EDI=\;\frac{E_{F}\times E_{D}\times F_{IR}\times C_{f}\times C_{m}}{w_{ab}\times T_{A}}=10^{-3}$$

where *E_F_* is the exposure frequency (365 d/y); *E_D_* is the duration of the exposure and is the Mexican average life expectancy (75.5 y) [29]; *F_IR_* is the fish consumption rate in Mexico (32.88 g/dd) [30]; *C_f_* is a conversion factor (*C_f_* = 0.208) from wet weight to dry weight considering 79% moisture in fish tissue; *C_m_* is the metal concentration in fish tissue (μg g^−1^ dry weight); *w_ab_* is the average Mexican adult weight 75 kg; and *T* (= *E_F_* × *E_D_*) is the average exposure for non-carcinogens.

### 2.6. Non-Carcinogenic Risk (THQ)

The non-carcinogenic risk of each TM was evaluated based on the Target Hazard Quotient (*THQ*) [31], which calculates the relationship between the potential TM exposure (quantity of the TM in a given time) and the theoretical exposure, in which no adverse effects are expected. *THQ* results from the relationship between the *EDI* and the *RfD* (Oral Reference Dose in mg kg/bwd), in which *RfD* estimates daily exposure to which a human population could be exposed throughout its lifetime without symptoms or signs of health deterioration through the following equation [27]:

(4) $$THQ=\frac{EDI\;}{RfD}$$

Therefore, if the *THQ* < 1, the population should not be at risk of presenting adverse effects, but if the *THQ* ≥ 1, the study population may present non-carcinogenic effects.

#### Hazzard Index (HI)

*HI* is the sum of TMs’ *THQ* values and estimates the potential health risk linked to all the TMs found in the fillet of *B. polylepis*. If *HI* < 0 adverse non-cancer health effects over an exposure lifetime would not be present (Equation (5)):

(5) $$HI=\;{\sum_{I=1}^{n}THQ}_{i}$$

### 2.7. Carcinogenic Risk

The incremental probability of a person to develop any type of cancer (ILRC) [32] was calculated.

CSFs measure the relative potency of carcinogens due to oral exposure. The EPA has developed CSFs as a result of the quantitative evaluation of exposure (oral) to a carcinogen, calculating the increase in cancer cases in a population [33]. CSFs are based on assumed lifetime risk (LR), so differences in consumption and body weight in childhood do not need to be considered [34]. Using CSF and exposure data in mg/kgd, cancer risks are calculated. Cancer limits of *ILCR* for one or more heavy metals are <10^−4^ (Equation (6)):

(6) $$ILCR\;={EDI}_{TM}\times {CSF}_{TM}\;or\;{RfD}_{TM}$$

CSFs measure the relative potency of carcinogens due to oral exposure. The EPA has developed CSFs as a result of the quantitative evaluation of exposure (oral) to a carcinogen, calculating the increase in cancer cases in a population [33]. CSFs are based on assumed lifetime risk (LR), so differences in consumption and body weight in childhood do not need to be considered [34].

The sum of the individual TM ILCR values in *B. polylepis* represents the total cancer risk (*TCR*) [35] (Equation (7)):

(7) $$TCR=\sum_{I=1}^{n}ILCR_{i}$$

### 2.8. Statistical Analysis

One-way ANOVA and Kruskal–Wallis test for normal and non-normal distribution were performed, respectively. In the case of significant differences, post hoc Tukey HSD (normal distribution), and Conover, Dunn, and Nementi (non-normal distribution), were performed. A Person’s test was performed to determine correlation [36] among TM concentrations; TM concentrations compared with weight, size, and collecting seasons were presented as mean (mg/kg^1^d) and standard deviation.

## 3. Results

### 3.1. Trace Metal Concentrations

The size and weight of the fishes showed a normal distribution ranging from 13.0 to 29.50 cm and 60 to 430 g, respectively. Person’s test showed a correlation between size and TM concentration (Pearson α = 0.05; *p* = 0.0169). Weight and TM concentration showed no correlation (Pearson α = 0.05; *p* = 0.079). The concentrations among trace metals and TM concentration among sampling period have not shown a normal distribution (Kolmogorov–Smirnov normality test: α = 0.05, KS = 0.277873; *p* < 0.01).

The TM concentration sequence was Zn > Fe > Mn > Cu = Pb > Ni > Cd.

The average, maximum and minimum concentration (mg/kg ± SD) were described for each TM. Mn, Zn, Cd, and Pb were the TM concentrations that exceeded the different TM maximum residues limits (MRLs) established by different agencies (Table 2).

**Figure 2:**
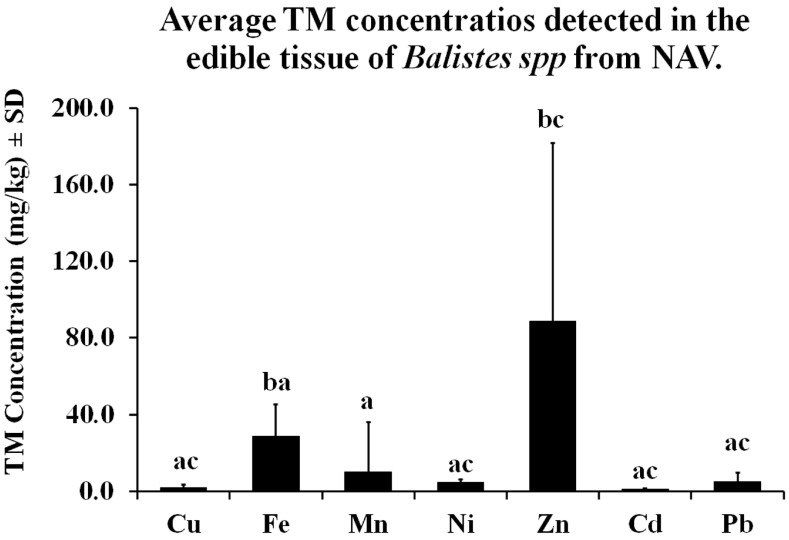
Average TM concentration ± SD detected in the fillet of *Balistes* spp. from the RAMSAR site 1826 San Ignacio-Navachiste-Macapule Lagoon Complex (NAV). Different letters mean significant differences among TM concentrations.

After Kruskal–Wallis analyses, a significant highest Zn concentration was described (α = 0.05), while the rest of the TM concentrations showed non-significant differences (Figure 2).

After Kruskal–Wallis statistical analysis (α = 0.05) among sampling periods with FE and Zn concentrations, Fe showed significantly different concentrations during December 2017. The lowest TM concentration was in winter 2018 (Figure 2). The highest concentrations of TM were in summer, coinciding with the period between July and October, when 50–85% volume precipitation occurs [41], and extraordinary floods have been recorded [42,43], which increase the mineral enrichment of waters, sediments, and suspended organic matter in the NAV.

**Figure 3:**
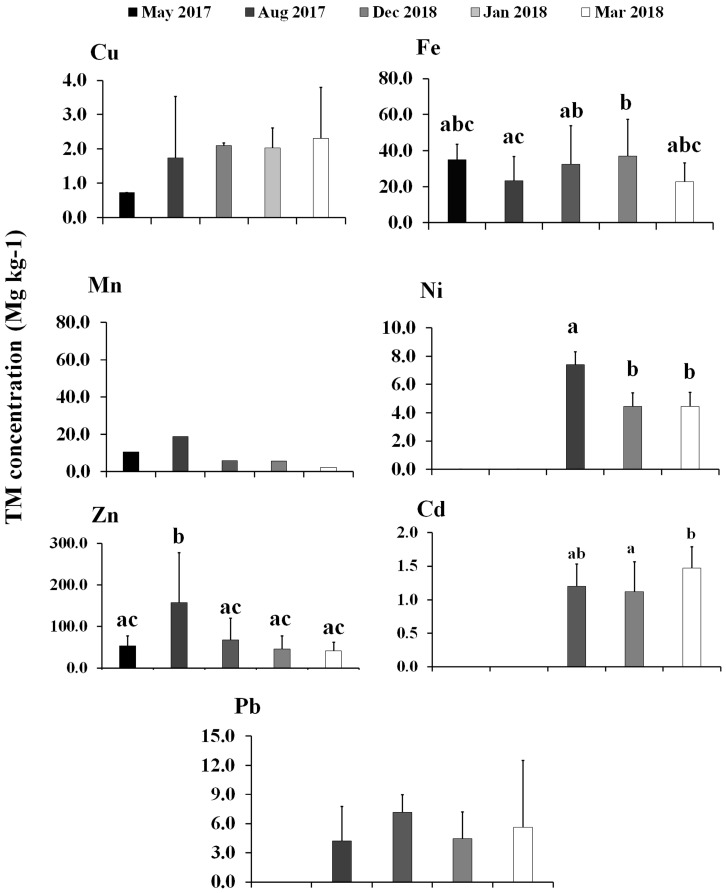
Seasonal TM concentrations in the RAMSAR site 1826 San Ignacio-Navachiste-Macapule Lagoon complex, Mexico. Zn and Fe have not exceeded the MRLs. Mn, a metabolic vital TM, was above MRLs. Cu, an essential TM, Cd, and Ni, a non-essential TM, were below MRLs, while Pb, a toxic TM, was above MRLs [44]. Different letters mean significant differences among TM concentrations.

By collecting periods, Cd showed significantly (*p* < 0.05) different concentrations among seasons, while for Zn the highest significant concentrations were in August 2017. Cu, Mn, Ni, Cd and Pb showed no significant differences among the collecting periods (Figure 3).

### 3.2. Estimated Daily Intake

The Zn and Cd concentrations in *B. polylepis* were above RfD values. The rest were below RfD concentrations.

### 3.3. Non-Carcinogenic (THQ) and HI

In the present study, Pb, Ni, Cd, Fe, Zn, and Cu THQ < 1, except for Zn (1.329), Cd (1.752), and Pb (1.577). On the other hand, Hi > 1 indicates adverse non-cancer health effects including all THQs of the TM over the exposure lifetime (Table 3).

### 3.4. Carcinogenic Risk

Zn and Mn results showed carcinogenic potential ILRC (5.72 × 10^−3^ and 1.2 × 10^−1^, respectively), indicating a high probability to develop cancer by ingesting the fillet of *Balistes* spp. In the present study, the mean ILCR values for Ni (4.02 × 10^−5^), Cd (1.58 × 10^−4^), and Pb (1.85 × 10^−4^) were under acceptable limit (1.0 × 10^−4^). The mean ILCRs values ranked Zn > Mn > Pb > Ni > Cd > Cu > Fe (Table 4).

## 4. Discussion

Based on the consumption rate of 32.88 g day^−1^, the concentrations of the detected TMs in the fillet were below the MRLs recommended by international agencies. The carcinogenic risk of Zn detected in *B. polylepis* (ILRC = 0.12) represents the highest cancer probability beside Mn and Zn ILRCs (5.72 × 10^−3^ and 1.12 × 10^−1^, respectively) detected in the fillet of *B. polylepis*. Compared to other TM concentrations described in previous reports, the present study’s Cu, Fe, Ni, and Pb concentrations (1.39, 25.39, 4.51, and 0.14 mg/kg, respectively) were among the lowest concentrations compared to other demersal fish. In the case of Mn, Zn, and Cd (4.51, 87.36, and 1.28 mg/kg, respectively), they were among the highest concentrations compared to other demersal fish (Table 5). The non-essential TMs such as Mn, Cd, and Pb are considered toxic, with values above MRLs, due to their metal properties, such as lipophilicity, chemical affinity to chelants, and metal ions with endogenous to large macromolecules [45]. Some of the most important anthropogenic sources of these metals’ residues are aquaculture and agriculture activities. More than 10,000 ha of shrimp aquaculture area surrounds the NAV site [20]. In the present study, shrimp aquaculture has been previously as described as a significant source of TM residues [46]. The high organic carbon, nitrogen and phosphorus content described in the sewage effluents rich in organic matter due to the residues of uneaten food in aquaculture [47,48], which sink in acid sediments, is correlated with the TM bioavailability [49].

Compared to other TM concentrations described in previous reports, the present study’s Cu, Fe, Ni, and Pb concentrations (1.39, 25.39, 4.51, and 0.14 mg/kg, respectively) were among the lowest concentrations compared to other demersal fish. In the case of Mn, Zn, and Cd (4.51, 87.36, and 1.28 mg/kg, respectively), they were among the highest concentrations compared to other demersal fish (Table 5). The non-essential TMs such as Mn, Cd, and Pb are considered toxic above MRLs due to their metal properties, such as lipophilicity, chemical affinity to chelants, and metal ions with endogenous to large macromolecules [45]. Some of the most important anthropogenic sources of these metals’ residues are the aquaculture and agriculture activities. More than 10,000 ha of shrimp aquaculture areas surrounds the NAV site [20]. In the present study, shrimp aquaculture has been previously described as a significant source of TM residues [46]. The high organic carbon, nitrogen and phosphorus content described in the sewage effluents rich in organic matter due to the residues of uneaten food in aquaculture [47,48], which sink in acid sediments, is correlated with the TM bioavailability [49].

**Figure 4:**
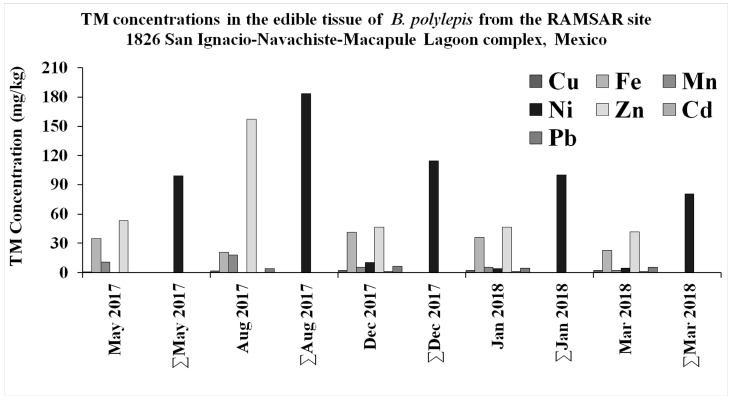
Seasonal TM and ∑TM per collecting period in *B. polylepis* from the RAMSAR site 1826 San Ignacio-Navachiste-Macapule Lagoon complex, Mexico.

Zn has shown the highest concentrations compared to other demersal fish previously studied (Table 5). In addition, its ILRC (1.2 × 10^−1^) gives it the highest risk of inducing cancer among the elements analyzed in this study. Zn is included as part of foliar spraying and nano-fertilizer to improve crop quality and yield of maize cultivars [58,59]. According to previous reports, high Zn concentration could be attributed to the intensive application of zinc sulfate ZnSO_4_ herbicide by farmers, and cow production [60]. The large use of fertilizer in the neighboring agriculture of the Guasave Valley area represents a giant source of Zn. Clay soil dominates the agriculture of Guasave Valley. Present intensive land use and current agriculture practices allow the concentration of these compounds in agricultural soils [61], where Zn residues could be discharged directly to the NAV aquatic ecosystem. When the ∑TM and individual TM concentrations were analyzed together, they were higher during the rainy season in the region between July and October [62,63] (Figure 4). During the rainy period, a hypereutrophic state has been reported in the Navachiste Lagoon System, with high productivity and very high trophic conditions, caused by the constant effluent of the drains that discharge in this ecosystem [22]. Zn as a micronutrient is involved in protein synthesis and nucleic acids, several enzyme cofactors, energy production, cell division and immunity [64]. In fish their accumulation has been reported through the gills and digestive track in the following order: liver > kidney > intestine > gill > muscle [65]. Previous reports have described that high Zn concentration (as in the present study) could be attributed to the intensive application of zinc sulfate ZnSO_4_ herbicide by farmers and the cow production [60,66]. Nevertheless, its excess in fish has been correlated with eosinophil DNA damage and cortisol [67], disruption of homeostasis, uncontrollable formation of ROS (Fenton reaction), overload-induced stress and hematology, histology, and immune response alterations [68].

The Fe concentrations in this study agree with species such as *Merlangius merlangus* and even with previously studied species in the NAV [10,52]. Iron sulfate FeSO_4_ is used as foliar fertilizer [69], the residues of which increase Fe concentration in soils. This Fe residue is related to runoff from the irrigation [70] in the Guasave Valley area which has been drained to the NAV. Other sources of FE could be the various metal component residues of brake pads from vehicles [71], which, due to wind transport, could be deposited in the NAV. In the cities, the main source of emissions of metal particles is vehicular traffic [72], and the density of automobiles in Guasave city has been increasing in the last 15 years, and in 2023 more than 58,000 vehicles were registered [73]. In addition, Fe is abundant in the continental crust, another source of this element, and the bioavailability concentrations in the sediments increase [74].

Mn is another element that is used as fertilizer in agriculture systems [69] and in shrimp aquaculture, and it is used as potassium permanganate KMnO_4_ as antibiotics [75,76]. After dietary absorption, it is a micronutrient in animal and plant nutrition. Its deficiency can provoke serious reproductive and skeletal abnormalities in mammals, but participates in the activation of hydrolases, kinases, decarboxylases, and transferases, and it is a vital component of the SOD, pyruvate carboxylase, glucose metabolism, and mitochondrial functioning. Another essential element, Cu, is crucial for DNA synthesis, cytochrome c oxidase of the electron transport chain of the antioxidant defense system, deposition in the yolk syncytial layer, enzyme lysyl oxidase, glucose metabolism and mitochondrial functioning, modulating levels of neutrophils and lymphocytes [27,77,78,79]. Multiple interactions among these essential TMs have been described, which exhibit competitive and cooperative models that depend on the chemical origin of the metal, the organ or tissue analyzed, the metal concentrations, and exposure to metals. The interaction may vary from antagonistic to synergistic or additive based on the quantity of metals in the mixture [80]. Mn-FE (oxyhydro)oxides release Cd under acidic soils and play an important role in Cd mobilization during drainage of residual waste from agricultural areas [81].

Pb IRLC of (5.3 × 10^−5^) means no risk to human health based on the consumption of *B. polylepis* with the resulting Pb concentrations (1.39 mg kg^−1^) in this study. Pb is an element that is considered toxic above very low concentrations (5 × 10^−3^ mg kg^−1^). Automobile emissions and industrial discharges are the most frequent sources of contamination. Guasave city is not industrialized, and primary and secondary economic activities are the most important. The population density is 98.29 inhabitants per Km^2^. In Sinaloa, Mexico, where NAV is located, the agroindustry is one of the main sources of lead exposure [82], and other sources not so frequently reported are the lead weights used in the artisanal fisheries [83], as usually occurs in NAV. Among the demersal species compared, *Sphoeroides* spp. and *B. polylepis* (this study) from the NAV showed the highest concentrations of Pb (Table 5). In the NAV, the seasons and areas of artisanal fishing—the main activity—have remained unchanged for decades, and the accumulation for decades of lost lead weights could be a significant source of contamination in these areas. Another Pb source is antifouling paints on vessels [74]. In general, artisanal fishermen use these Pb base paint products to prevent the attachment of marine organisms such as barnacles to their vessels [84]. Currently, there are more than 1500 small vessels (SVs) registered and operating in NAV [85]. The NAV covers almost 80,000 ha of coastal waters [21], and this number of small vessels (sv) represents a density of 0.02 sv/ha, but when their fishing operations become restricted to the traditional fishing zones only (<32,000 ha) [86], it increases to 0.05 sv/ha. The constant maintenance of this fleet during each fishing cycle, which consists of scraping and removing the old antifouling paint without regulation in the fishing towns, represents a source of lead contamination. In fish, the excessive Pb in their tissues can decrease T-AOC and GSH levels and the activity of GPx, SOD, and CAT, increasing lipid peroxidation, which indicates the antioxidants’ inability to eliminate free radicals and their depletion [68].

Ni concentration (4.67 mg/kg) detected in the present study was similar to other demersal species such as *Merlangius merlangus* and *Mycteroperca olfax* [52,55]. *B. polylepis* is a species that inhabits sandy-muddy bottoms or muddy bottoms and its TM concentrations were similar to previous reports concerning species living in the same ecosystem. Ni concentrations up to 30 times higher have been reported in *Mullus murmuletus*, *Pagellus erythrinus, Merluccius merluccius* [56], and *Caulolatilus princeps* [55], which could be related to rainfall runoff, which carries TM residues that are then transported and deposited in the marine ecosystems [87,88]. The constant contamination of coastal ecosystems by agricultural wastes or aquaculture feeds has been reported as a source of TM [89] and the neighboring Guasave agriculture valley has been described as a source of pesticide residues [90]. Ni base antifouling or anticorrosive paints used in aquaculture, coastal infrastructure and vessel maintenance is another potential source of Ni [84]. TM accumulation is associated with fine, muddy sediment, and could be trapped in sediments in semi-enclosed water bodies with a long water retention time [91], as could be occurring in NAV. *B. polylepis*, as a generalist predator, could incorporates these TM residues previously bioaccumulated by the mollusks, crustaceans, and organisms it feeds on [6].

Cu base substances are used in shrimp aquaculture and agriculture sectors, which negatively impact the coastal ecosystems of NAV, and represent an intermittent pollutant element that constitutes copper-based foliar fertilizers and pesticides for agriculture [70,92] and in shrimp aquaculture such as algicide, bactericide, and fungicide [93,94]. Higher Cd concentrations in aquatic ecosystems are closely related to regions significantly impacted by anthropogenic activities. Higher Zn and lower Cd concentrations are related to the physiological competence of the active site of Zn by Cd, and it is possible that metallothionein could be binding the excess of Cd to protect the organism against this xenobiotic metal through the detoxification-rescue system (Cd_n_Zn_(7−n)_MT) [95]. In the present study, Zn levels were nearly 76 times greater than Cd, which may indicate a substantially higher bioavailability of Zn in the aquatic environment of NAV. A much higher concentration could favor Zn for active sites on proteins, enzymes and other organic molecules in *B. polylepis*.

## 5. Conclusions

Based on the results of TM ingested through the fillet of *B. polylepis* from NAV, and considering the fish consumption rate in Mexico (32.88 g day^−1^), this species represents a human health risk due to the development of cancer potentially caused by its high Zn and Mn content. In the fillet of *B. polylepis*, Zn and Mn, even though they are considered micronutrients with biological functionality, were found at carcinogenic concentrations and higher EDI values. The TMs showed significant different concentrations among them, but Zn showed up to 76 times higher concentration than the rest of the elements. The omnivorous diet of *Balistes* spp., based on sea urchins, small crustaceans, mollusks, detritus, algae, and coral rocks, could explain the variety of TM concentrations found in the fillet, due to the bioaccumulation and trophic transfer rates previously described for aquatic food webs. The elements analyzed in this work are highly lipophilic, although it is necessary to determine the pollution degree in NAV and the health risks due to the consumption of marine species from this lagoon, including the TM concentrations in species within the food web, sediments, and water, as well as their dispersion through the food web. TM bioaccumulation rate, translocation, and information regarding water pH and temperature can help in determining the TM bioaccumulation rate in this species. Health risk analysis should include social aspects. It is essential to conduct well-founded surveys to collect information regarding the human consumption habits of marine organisms in the coastal areas of NAV. It is vital to obtain population data for a more reliable and significant estimation of health risk. In addition, it is recommended to carry out GIS spatial mapping including TM data for monitoring, and the promotion of health awareness for management and treatment options.

## Figures and Tables

**Table 1:** Average concentration (mg/kg) of the certified reference material TORT-3 (lobster hepatopancreas Certified Reference Material).

Element	Mass Fraction (mg/kg)
Cd	42.3 ± 1.8
Cu	497 ± 22
Fe	179 ± 8
Mn	15.6 ± 1.0
Ni	5.30 ± 0.24
Zn	136 ± 6
Pb	0.225 ± 0.018

**Table 2:** Average, maximum, minimum, and standard deviation TM concentrations (mg/kg ± SD) detected in the fillet of *Balistes* sp. from the RAMSAR site 1826 San Ignacio-Navachiste-Macapule Lagoon Complex. Cells in grey are TM concentration above MRLs.

TM	MRLs	Average	Maximum	Minimum	SD
Cu	5 *–30 **	1.39	9.62	0.16	1.38
Fe	-	25.47	84.32	6.15	28.47
Mn	0.14 ****	8.98	238.38	0.10	26.14
Ni	70 **	0.38	10.59	2.097	1.54
Zn	0.3 ***	87.53	656.30	11.33	92.48
Cd	0.003 ****	1.151	2.17	0.30	0.41
Pb	0.30 *	1.23	31.45	0.64	4.72

* [37], ** [38], *** [39], **** [40].

**Table 3:** Estimated daily intake (EDI), concentration of non-carcinogenic hazard quotient, and carcinogenic hazard quotient in the fillet of *Balistes* spp. from the RAMSAR site 1826 San Ignacio-Navachiste-Macapule Lagoon Complex (NAV).

Metal	Average Concentration (mg/kg)	Reference Dose (RfD)	EDI	THQ	HI
Cu	1.39	NR	0.006		2.9
Zn	87.53	0.003 *–0.3 **	0.399	1.329	
Ni	0.38	0.02 *	0.002	0.101	
Fe	25.47	NR	0.116		
Mn	8.98	0.14 *	0.041	0.292	
Cd	2.17	0.0004 *–0.05 **	0.005	1.752	
Pb	1.23	0.30 *	0.022	2.577	

NR = non-regulated. * [40], ** [37].

**Table 4:** TM Estimated Daily Ingest (EDI), TM Reference daily dose, and incremental lifetime cancer risk (ILRC). Grey cells indicate ILRC above maximum cancer limits (1 × 10^−4^).

TM	EDI	RfD	ILRC
Cu	0.006	NR	
Zn	0.399	3 × 10^−1^	1.2 × 10^−1^
Ni	0.002	2 × 10^−2^	4.02 × 10^−5^
Fe	0.116	NR	
Mn	0.041	1.4 × 10^−1^	5.72 × 10^−3^
Cd	0.001	3 × 10^−3^	1.58 × 10^−4^
Pb	0.006	3 × 10^−3^	1.85 × 10^−4^

**Table 5:** Comparison of TM concentrations in *Balistes* spp. the RAMSAR site 1826 San Ignacio-Navachiste-Macapule Lagoon Complex (NAV) to other fish content studies (mg/kg).

Species	Cu	Fe	Mn	Ni	Zn	Cd	Pb	Habitat [17]
*Balistes polylepis* *	*1.39*	*25.39*	*8.95*	*0.44*	*87.36*	*1.15*	*1.39*	A
*Amblychaeturichthys hexanema* ^3^	**7.9**				71.7	0.45		A
*Caulolatilus princeps* ^7^				1.19		0.39	0.44	A
*Chelidonichthys lucerne* ^2^						0.003	0.04	A
*Collichthys lucidus* ^9^	2.8				15.15	0.067	0.1	A
*Conger myriaster* ^3^	**4.78**				29.8	0.22		A
*Cynoglossus joyneri* ^3^	6.95				44.9	0.36		A
*Hexagrammos otakii* ^3^	**5.81**				**60.8**	**0.19**		A
*Johnius belangerii* ^3^	4.66				67.4	0.38		A
*Larimichthys crocea* ^9^	2.0				12.55	0.067	0.025	B
*Larimichthys polyactis* ^3^	5.9				56.1	0.39		B
*Lateolabrax maculatus* ^9^	5.1				12.85	0.067	0.1	C
*Lepturacanthus savala* ^3^	4.752				36.6	0.28		C
*Merlangius merlangus* ^2^						0.003	0.04	D
*Merlangius merlangus* ^4^	0.5	0.87	0.93	0.01	3.4	0.02		D
*Merlangius merlangus* ^4^	1.32	**98.1**	0.16	0.135	65.4	0.21	0.53	D
*Merlangius merlangus* ^6^						0.0055	0.0064	D
*Merluccius merluccius* ^8^	3.16			7.69	33.77			E
*Miichthys miiuy* ^3^	2.7				53.4	0.22		E
*Mullus barbatus* ^4^	0.5	2.3			3.2	0.02	0.05	E
*Mullus barbatus* ^5^						0.00249	0.13	E
*Mullus surmuletus* ^8^	5.31			15.13	31.99			A
*Muraenesox cinereus* ^3^	4.16				14.5	0.14		E
*Muraenesox cinereus* ^9^	2.3				**21.4**	**0.068**	**0.1**	E
*Mycteroperca olfax* ^7^				0.48		2.54	0.25	F
*Pagellus erythrinus* ^8^	2.54			12.14	34.29			F
*Saurida elongate* ^3^	7.43				82.9	0.48		F
*Sebastiscus marmoratus* ^3^	3.4				94.5	0.15		E
*Sebastiscus marmoratus* ^9^	2.4				27.15	0.067	1.1	E
*Sphoeroides* spp. ^1^	1.45	80.52	6.13	8.06	189.55	1.45	18.42	E

ND = Below Limited Detection. * Present study. A = Sandy-muddy bottoms, B = Sand, muddy sand or gravel bottoms, C = Rocky, soft sand and mud bottoms, D = Rocky coastal areas, E = Mud, gravel, sand and rock bottoms, F = Coastal waters and estuaries depth > 70 m); ^1^ [10] ^2^ [50] ^3^ [51] ^4^ [52] ^5^ [53] ^6^ [54] ^7^ [55] ^8^ [56] ^9^ [57]. Bold letters indicate the Maximum TM detected among species. Italics letters correspond to the present study results.

## Data Availability

The raw data supporting the conclusions of this article will be made available by the authors on request.
